# Konjac Glucomannan Induced Retarding Effects on the Early Hydration of Cement

**DOI:** 10.3390/polym14051064

**Published:** 2022-03-07

**Authors:** Yushan Chen, Pengfei Tang, Chen Zhong, Laibao Liu, Yunsheng Zhang, Youhong Tang, Hongping Zhang

**Affiliations:** 1 State Key Laboratory of Environmentally Friendly Energy Materials, School of Materials Science and Engineering, Southwest University of Science and Technology, Mianyang 621010, China; cys_2687@163.com (Y.C.); pf_tang@163.com (P.T.); z15508003605@163.com (C.Z.); liulaibao@swust.edu.cn (L.L.); 2 College of Civil Engineering, Lanzhou University of Technology, Lanzhou 730050, China; zhangyunsheng2011@163.com; 3 Institute for NanoScale Science and Technology, College of Science and Engineering, Flinders University, Adelaide, SA 5042, Australia

**Keywords:** konjac glucomannan, setting time regulator, hydration kinetics, interaction mechanism

## Abstract

Customarily, retarders serve as the setting time regulators of cement-based composites to meet the demands of various construction environments. However, the limited ability to adjust the setting time restricts the application of polysaccharides in special environments. In this study, we reported a naturally high-efficiency retarder, konjac glucomannan (KGM), and studied the mechanism of its effect on the hydration of ordinary Portland cement. Incorporating KGM could significantly prolong cement hydration without strength damage. Furthermore, the active hydroxyl group (−OH, rich in KGM) could chelate with Ca^2+^ (released from cement hydration) to form a cross-linking network, which is adsorbed on the surface of cement clinker, thereby being conducive to delaying the process of cement hydration and reducing the heat of hydration. The findings of this study are critical to the ongoing efforts to develop polysaccharide-cement-based composite materials for application in various special environments.

## 1. Introduction

Generally, sugars serve as the setting time regulator to increase the workability time of cement-based composites, which simultaneously obtain other properties, such as water retention [1,2], ease of spraying [3,4], and antisegregation [5,6]. To date, various polysaccharides—such as starch [7,8], cellulose [9,10], and xanthan gum [11]—have been applied in cement. However, the limited ability of the polysaccharides to adjust the setting time makes it unable to meet all needs of various construction environments, such as bridge pouring, mass concrete building, and excess concrete treatment. Therefore, it is urgent to develop an admixture that efficiently modifies cement-based composite setting time.

Until now, numerous studies have been conducted to reveal the regulation mechanisms of various polysaccharides. The retardation of maltodextrin on cement was studied by Lei et al. [12]; they showed that the maltodextrin molecular layer that adsorbs on the cement surface inhibits the hydration process. Furthermore, Wang et al. [13] discovered that α-hydroxy O, α-carboxylate O, and β-carboxylate O on citrate ligands coordinating with [Mg(OH)(H_2_O)_x_]^+^ form a stable trinuclear positive complex [Mg_3_(OH)_10−x_(Hcit)(H_2_O)_x_]^+^ (Hcit: the citrate ligand). The formed complex restricts the reaction of [Mg(OH)(H_2_O)_x_]^+^ with OH^−^, hinders exposure and continuous hydration of fresh MgO, and increases the setting time of magnesium oxysulfate cement and MgO content. However, as reported by Alexander et al. [14], alginates act to accelerate the hydration of calcium aluminate cement (CAC) with chelate Ca^2+^ as well. It is indicated that active functional groups in polysaccharides, such as hydroxyl and carboxyl groups, will adjust the setting time of cement through Ca^2+^ chelation. Although diverse adjusting mechanisms of polysaccharides have been reported, the interaction mechanisms of polysaccharides and cement-based composites are still controversial, and further studies on extensive regulation effects of polysaccharides on the hydration process of cement-based composites are needed.

Konjac glucomannan (KGM) attracts our attention as it is rich in actively functional groups (−OH) [15]. KGM comprises β-d-glucose and β-d-mannose at a molar ratio of 2:3 or 1:1.16, which the molecular chain is formed by β-1,4 glycosidic bonds in the main chain and β-1,3 glycosidic bonds in the side chains [16,17]. Owing to its excellent chelating ability on metal ions, KGM is often used in the field of environmental treatment to adsorb heavy metal ions, such as Pb(II) [18,19], Cd(II) [20], and Cu(II) [21]. In addition, the abundant functional groups (hydroxyl) endow KGM with excellent gelation [22,23], thickening [17,24], beneficial effects on syneresis [25,26], and other characteristics [27,28,29]. Moreover, KGM is a natural polysaccharide extracted from konjac with advantages of wide sources, low price, and easy availability. In summary, KGM is expected to be a potential admixture candidate to adjust the setting time of cement.

Thus, KGM is used as a cement admixture to explore its effects on cement hydration kinetics and thermodynamics systematically. At first, to elucidate the regulation of setting time, various experiments on the setting time of ordinary Portland cement (OPC), calcium sulfoaluminate cement (CSA), and CAC with different KGM contents were performed. In addition, the influence of KGM with different molecular weights on the hydration process of OPC was studied via isothermal heat flow calorimetry. Furthermore, the time-dependent hydration products were monitored by X-ray diffractometry (XRD) and Fourier transform infrared (FTIR) spectroscopy at an early stage of cement hydration. Finally, the interaction mechanism between KGM and cement was revealed through adsorption (total organic carbon) and ion chelation (inductively coupled plasma atomic emission). From the above experiments, we hope to deeply understand the effect of ion chelation via active functional groups on the setting time of cement to further understand the interaction mechanism between KGM and cement-based composites.

## 2. Materials and Methods

### 2.1. Materials

OPC (42.5 R), with a density of 3.04 g/cm^3^, was purchased from Sichuan Shuangma Cement Co., Ltd., Mianyang, China. CAC and CSA were purchased from Jiuqi Building Material Co., Ltd., Zhucheng, China. All types of cement were used without any admixtures, and their oxide composition (Tables S1–S3 in Supporting information) was determined using X-ray fluorescence (Axios, PANalytical, Almelo, The Netherlands). KGM with viscosity ≥ 15,000 mPa·s was purchased from Yuanye Biological Co., Ltd., Shanghai, China, and used without further purification. KGM with different molecular weights was obtained via “heterogeneous hygrothermal degradation treatment” [30]. A conical flask containing KGM powder was placed in a vertical autoclave (YXQ-LS-18SI, Boxun Co., Ltd., Beijing, China) and treated for 60 and 20 min at 121 °C, which were denoted as KGM-1 and KGM-2, respectively. Their apparent viscosity was determined by an NDJ-8S rotary viscometer, whereas the viscosity average molecular weight was investigated by an Ubbelohde viscometer (Table S4 in Supporting information).

### 2.2. Sample Preparation

To prepare cement pastes, various contents of KGM (0, 0.01, 0.02, 0.05, 0.1, 0.2, and 0.5 wt %) were dissolved in deionized water for 24 h. Then, the cement pastes with a water/cement ratio (W/C) of 0.5 were stored in sealed plastic cups to prevent carbonization and dehydration. The environmental temperature was kept at 20 °C ± 1 °C for curing. To fabricate the cement mortars (W/C = 0.5), stable and uniform solutions with KGM contents of 0%, 0.01%, 0.02%, 0.05%, 0.1%, and 0.2% were obtained. Then, the cement and sands with a ratio of 1:3 were mixed with prepared KGM solutions. The precast cement mortars are cured within molds at 20 °C and at a relative humidity of 70% for 2 days before being demolded.

### 2.3. Experimental Methods

#### 2.3.1. Setting Time

The normal consistency for OPC, CAC, and CSA was measured as W/C = 0.28 according to the ASTM C191 standard. The setting time was measured by the Vicat apparatus (SS-S-403, Shinohara Manufacturing Co., Ltd., Shizuoka, Japan). All measurements were repeated 3 times.

#### 2.3.2. OPC Workability Characterization

To determine the workability of OPC mixed with KGM, a fluidity test was performed according to the ASTM C1437 standard. The fluidity of the cement paste is the average of the largest diameters in two vertical directions, and each value is measured three times. Meanwhile, the viscosity of cement paste under different KGM contents was tested with an NDJ-8S rotary viscometer, where results were repeated three times. Compressive strength was measured according to the ASTM C109M standard. The universal mechanical testing machine (Instron 5567, Boston, MA, USA) was used for sample compressive strength measurement, and the measurements were repeated 6 times.

#### 2.3.3. Microstructure of Cement Mortar

A porosity experiment was performed using the static nitrogen adsorption instrument (W-BK122, Jingwei Gaobo, Beijing, China) on cement mortar samples after the hydration was stopped with the solvent exchange reaction. Moreover, the microscopic morphology of cement mortar (about 0.2 g for each) was characterized by a high-resolution field emission scanning electron microscope (HRFE-SEM, Zeiss Ultra55, Oberkochen, Germany).

#### 2.3.4. Cement Paste Hydration Heat

The heat release process of cement was measured by a cement hydration heat tester (TAM AIR, Worcester China Co., Ltd., Shanghai, China), with a sensitivity of ±20 μW and detection limit of 4 μW. All tests were performed at a constant temperature of 20 °C ± 0.02 °C, and the W/C was set to 0.5 (about 5 g cement for each) to ensure good fluidity of the cement paste and good KGM dispersion.

#### 2.3.5. Hydration Product Characterization

An X-ray diffractometer (TD3500, Dandong Tongda Technology Co., Ltd., Dandong, China) was used to examine the phase development in cement pastes blended with different dosages of KGM on the operating of 2θ between 5° and 70° at 30 KV and 30 mA. The interaction of KGM with hydration products and ions in the pore solution were observed via FTIR (Spectrum Two+ Frontier, Perkinelmer Co., Ltd., Waltham, MA, USA) in the scanning range of 400–4000 cm^−1^.

#### 2.3.6. Interaction of KGM and Cement Characterization

A Vario total organic carbon (TOC) cube instrument (XPERT-TOC/TNb, Trace Elemental Instruments, The Netherlands) was used to measure the amount of KGM remaining in the cement pore solution that did not adsorb on the surface of the cement particle. The inductively coupled plasma atomic emission (ICP-OES) spectroscopy (Agilent Technologies, Palo Alto, CA, USA) was performed to measure the concentrations of Ca^2+^, Al^3+^, and ${\mathrm{SiO}}_{3}^{2-}$ in the cement pore solution with different KGM dosages. 

20 g cement, 10 g deionized water (W/C = 0.5), and KGM with different dosages were mixed and filled into a 50 mL centrifuge tube. After curing for 15 mins, the samples were centrifuged at 5000 rpm for 2 mins and then diluted to a proper concentration. All results were repeated three times.

#### 2.3.7. Molecular Dynamics Simulations 

The KGM molecular chain was built and immersed in a water box with the size of 36.7 Å × 35.7 Å × 36.7 Å. Then, the KGM water solution model was added onto C_3_A (100) and C_3_S (001) surfaces separately. All the molecular dynamics (MD) simulations were carried out by Forcite in MaterialsStudio 4.0 software, and the CVFF force fields were used. In detail, the 200 ps MD simulations are performed under the NVT ensemble with a time step of 0.1 fs. Then, the interactions of the systems were analyzed according to the MD trajectory. 

## 3. Results and Discussion

### 3.1. Effect of KGM on the Setting Times

**Figure 1:**
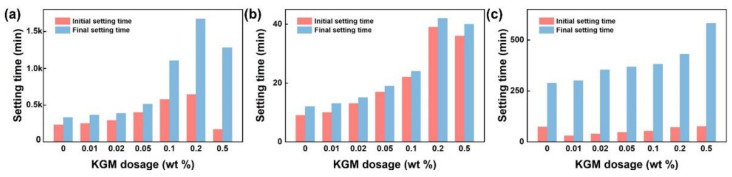
(**a**–**c**) The setting times of OPC, CSA, and CAC with different KGM dosages (W/C = 0.28).

At the very beginning, KGM was found to be the retarder of cement, where the setting time could be extended by adding various contents of KGM. As shown in Figure 1, both the initial and final setting times of OPC and CSA were prolonged with the addition of KGM dosage rising from 0 to 0.2 wt %. From Figure 1a, with the addition of KGM, the initial setting time of OPC increased from 230 min (the paste) to 250 min (0.01 wt %), 400 min (0.05 wt %), and 640 min (0.2 wt %), whereas the final setting time ranged from 330 min to 365 min (0.01 wt %), 510 min (0.05 wt %), and 1670 min (0.2 wt %). Furthermore, as shown in Figure 1b, the initial setting time of CSA was just 8 min, whereas it ranged from 10 to 40 min with the different KGM dosages (0.01–0.2 wt %). This phenomenon illustrates that KGM is an outstanding cement retarder, and the retardation is longer than that of various commercial retarders (see Table S5 in Supporting information [31,32,33,34,35,36,37,38]). Interestingly, an entirely different phenomenon appeared on CAC where KGM promoted and delayed the initial and final setting times of CAC, respectively (Figure 1c). The initial setting time reduced from 75 to 30 min with a certain KGM dosage (0.01 wt %); however, the initial setting time gradually ranged from 30 to 76 min with the growth of KGM content (in the range 0.01–0.5 wt %). Meanwhile, the final setting time of CAC was prolonged with the increase in KGM dosage. In other words, the accelerating of the initial setting time negatively correlated with the amount of KGM, whereas the retardation of the final setting time positively correlated with the KGM dosage.

**Figure 2:**
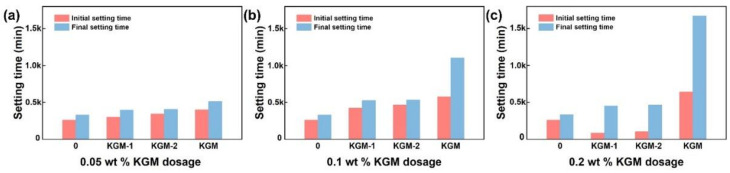
The setting times of OPC, CSA, and CAC with different KGM molecular weight. (**a**) with 0.05 wt % KGM; (**b**) with 0.1 wt % KGM; (**c**) with 0.2 wt % KGM.

In addition, different molecular weights of KGM were introduced to the OPC to further explore its regulation. As shown in Figure 2a–c, all molecular weights of KGM present a retarding trend where the setting time prolonged with the KGM content ≤0.1 wt %. Notably, the retardation significantly prolonged as the molecular weight of KGM increased. However, the setting time of KGM-1 and KGM-2 at 0.2 wt % showed the same trend with that of KGM at 0.5 wt %, which is that the initial setting time reduced, whereas the final setting time increased. These results indicated that excessive KGM dosages converse its effect from retarding to accelerating, as is also generally recognized for carboxylic acids, such as citric acid [39]. Therefore, we have reasons to conclude that KGM serves as a setting time regulator, and the setting time regulation depends on cement type as well as KGM molecular weight and dosage.

Furthermore, the influence of KGM on the mechanical properties of mortar was studied (see Figure S1 in Supporting information). The compressive strength variation of OPC due to the incorporation of KGM depends on its content and curing age of the mortar. As shown in Figure S1a–c, the compressive strength of mortar increased with the growth of the curing age. At different ages, the overall compressive strength of the mortar tended to be enhanced with the amount of KGM increasing. At 28 curing days, the compressive strength of mortar enhanced by about 6.26–17.16% at a certain KGM addition (0.1–0.2 wt %). To further understand the effect of KGM on the mechanical properties of mortar, the porosity ratio of mortar admixed with KGM at various dosages was characterized; the results are shown in Figure S1d,e. The cumulative pore volume of cement mortar at 28 days was 130 cm^3^/g of which with the distribution of pore size in the ranges <20, 20–50, and 50–200 nm accounted for 68.93%, 15.18%, and 15.89%, respectively. With the addition of KGM, the cumulative porosity volume of cement mortar increased from 0.130 to 0.150 cm^3^/g (0.02 wt %), 0.198 cm^3^/g (0.1 wt %), and 0.148 cm^3^/g (0.2 wt %). For mortars with certain KGM dosages (0.1 and 0.2 wt %), although the porosity volume increased, it was due to an increase in the harmless (<20 nm) or less harmful pores (20–50 nm), which was generally considered to insignificantly affect the compressive strength [40,41,42]. This phenomenon suggested that KGM could be an efficient retarder of OPC without compressive strength damage.

### 3.2. Hydration Kinetics of OPC with KGM

**Figure 3:**
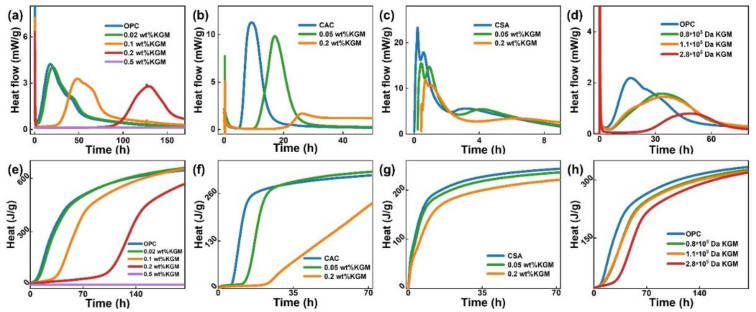
(**a**–**c**) Heat flow calorimetry of cement (OPC, CAC, and CSA) pastes admixed with various KGM dosages at W/C of 0.5; (**d**) Heat flow calorimetry of OPC pastes incorporated with different KGM molecular weights at W/C of 0.5; (**e**–**g**) Cumulative heat flow of cement (OPC, CAC, and CSA) pastes admixed with various KGM dosages at W/C of 0.5; (**h**) Cumulative heat flow of OPC pastes incorporated with different KGM molecular weights at W/C of 0.5.

To supplementary comprehend the influence of KGM on cement hydration, the hydration kinetics of various (OPC, CAC, and CSA) cements were studied by monitoring the heat evolution during their hydration process via the heat flow calorimetry at a W/C of 0.5. The schematic diagram of the cement hydration process and the effect of KGM were described in Figure S2 and Table S6 of Supporting Information. As is shown in Figure 3, the hydration processes of OPC, CAC, and CSA were all delayed with the addition of the KGM, and the larger the amount of KGM the more apparent the retardation effect. It is worth noting that the deadline of the induction period was extended (by 20-fold (OPC), 4-fold (CAC), and 2-fold (CSA) at 0.2 wt % content of KGM) and the acceleration period was accelerated (by seven-fold (OPC), three-fold (CAC), and two-fold (CAS) at 0.2 wt % content of KGM), respectively. In addition, the prolongation of the induction period and the slowing down of the exothermic rate of the acceleration period also indicated that the addition of KGM could hinder the nucleation and growth of hydration products. Besides, the heat release of cement hydration declined with the addition of the KGM, where the peak exotherm of the acceleration period decreased by 36.4% (OPC), 85.2% (CAC), and 38.5% (CSA) at 0.2 wt % (See Figure 3a–c), and the cumulative heat reduced by 12.0% (OPC), 27.1% (CAC), and 9.2% (CSA) at 0.2 wt %, individually (see Figure 3e–g). Furthermore, the molecular weight of KGM is critical for its retardation on cement hydration. The retardation and reducing the heat of hydration would be improved by increasing the molecular weight of KGM (See Figure 3d,h). Thus, KGM can be served as a universal retarder and temperature rise inhibitor (TRI) simultaneously. The performance was demonstrated by comparison with other admixtures (see Table S5 in Supporting information).

The retardation of KGM on cement hydration could be explained by the inhibiting nucleation and growth of hydration products and reducing the heat of hydration. There was no hydration exotherm detected (Figure 3a,e) at the 0.5 wt % KGM dosage, which indicated that the acceleration effect of excessive KGM dose on OPC is related to the thickening effect of KGM [43,44,45], which is regarded to significantly relate to its concentration [46]. The viscosity coefficient of the 0.5 wt % KGM solution was about 1200 and 12 times those of 0.1 and 0.2 wt % KGM solutions, respectively [34]. In addition, the result of cement fluidity with different KGM contents is that the cement fluidity decreased rapidly when the KGM content was more than 0.1 wt % (see Figure S3 in Supporting Information) further supported the hypothesis. To supplement the study of the retardation mechanism of KGM, OPC (the most widely used cement) was taken as an example to explore the interaction between KGM and cement.

### 3.3. Hydration Products of OPC with KGM

**Figure 4:**
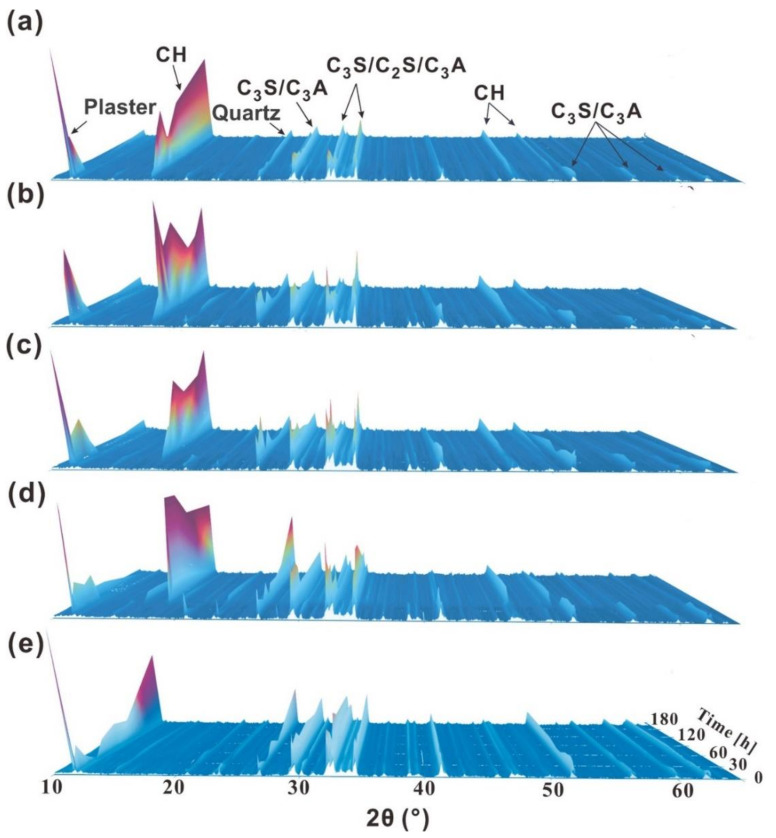
Hydration products of OPC with different KGM dosages (W/C = 0.5). (**a**) the paste without KGM, (**b**) with 0.02 wt % KGM, (**c**) with 0.1 wt % KGM; (**d**) with 0.2 wt % KGM; (**e**) with 0.5 wt % KGM.

To further probe into the retardation of KGM, XRD was employed to investigate the development of hydration products over time, which was closely related to the hydration process and specimens’ strength. As shown in Figure 4a, the peak of cement clinker (such as CaSO_4_) could be observed while there was no peak of the hydrated product (such as CH) in the early period of hydration. As the hydration reaction progressed, the peak of CaSO_4_ of the cement paste disappeared at about 8 h, whereas the peak of CH appeared at about 8 h. However, with the KGM dosage increase (0.02–0.2 wt %), the disappearance of the peak of CaSO_4_ extended to about 24 (0.02 wt %) and 36 h (0.1, 0.2 wt %), whereas the appearance of CH diffraction peak prolonged to about 24 (0.02 wt %), 36 (0.1 wt %), and 48 h (0.2 wt %, see Figure 4b–d). Moreover, the CH cannot be found in the hydration products with the 0.5 wt % KGM addition, which indicated no apparent hydration in the cement paste (Figure 4e). The above results were well supported by FTIR (see Figure S4 in Supporting Information), which also showed that the appearance of the stretching vibration peak of −OH (from Ca(OH)_2_, at about 3640 cm^−1^) prolonged with the addition of KGM.

Hydration products are intimately connected with the setting time of cement due to hindering the relative movement of water and particles to solidify cement with the generation of a large amount of hydration products. The results found here correspond well with those of Figure 3a,b and further confirmed that: (1) the incorporation of KGM will hinder the nucleation and growth of hydration products; (2) the acceleration effect caused by excessive KGM dose is a result of the thickening of KGM.

### 3.4. Interaction Mechanism of KGM and Cement

**Figure 5:**
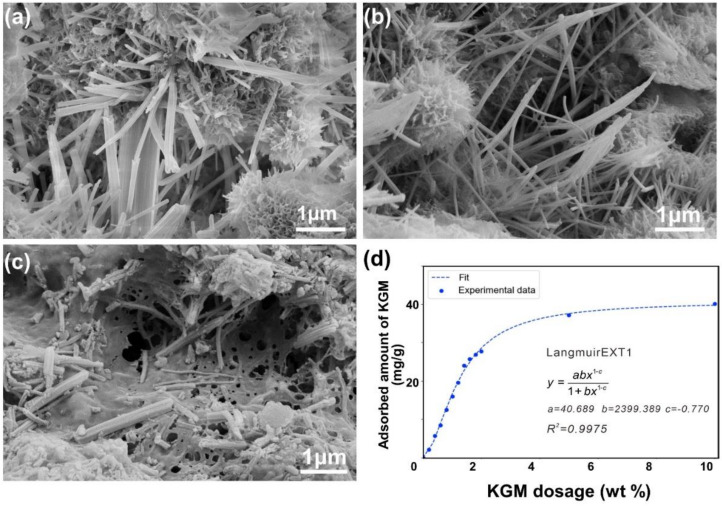
Morphology of hydration product changes with different KGM dosages: (**a**) the paste, (**b**) with 0.1 wt %, (**c**) with 0.2 wt %; (**d**) adsorption isotherm for KGM admixed to cement paste at W/C = 0.5.

To deeply understand the interaction mechanism between KGM and cement, various studies on the morphology, KGM concentration, and ion concentration in the pore solution—as well as the interaction between KGM and ions-were conducted; the results are shown in Figure 5. Similar to the reports of other polysaccharides [34,47], the morphology and quantity of the hydration products changed significantly with the addition of KGM. As shown in Figure 5a, the ettringite in the cement was flat and elongated. However, with the addition of KGM, the ettringite gradually became rod-shaped (Figure 5b,c). Surprisingly, at the early stage (1 day), a film-like substance was found to coat the surface of hydrated products and cement clinker in 0.2 wt % samples (see Figure 5c and Figure S5 in Supporting information). In addition, TOC was employed to further explore the composition of the film-like substance. As shown in Figure 5d, the amount of KGM adsorbed on cement paste increased with KGM dosage (<4%), which corresponded well with a Langmuir isotherm until it reached a plateau, referring to the saturated adsorption at approximately 27 mg KGM/ 1 g cement. This phenomenon indicated that the film-like substance is the KGM. That is, KGM would affect the morphology of hydration products by adsorbing on the surface of hydration products.

**Figure 6:**
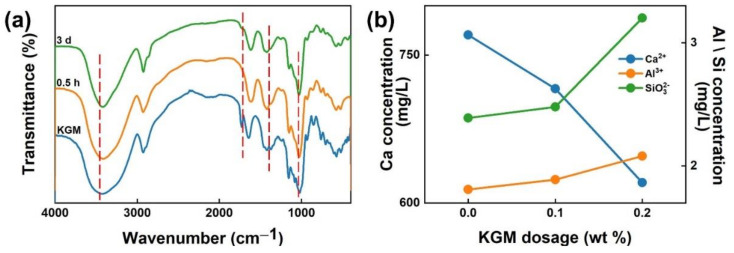
(**a**) KGM chelates metal ions in the pore solution of cement; (**b**) ion concentrations of Ca^2+^, Al^3+^, and ${\mathrm{SiO}}_{3}^{2-}$ dissolved in the pore solution of cement (unit: mg/L, W/C = 0.5) treated with different KGM dosages.

In addition, the interaction between KGM and cement pastes was further studied by the FTIR and ICP-OES analyses; the results are shown in Figure 6a,b. A broad intense peak at around 3435 cm^−1^ was observed on the FTIR spectrum of KGM, corresponding to the stretching of −OH; whereas the peak at around 1740 cm^−1^ matching the carbonyl was assigned to the acetyl groups in the KGM. The band in the region of 1645 cm^−1^ was relevant to the in-plane bend vibration of −HOH of KGM, whereas those in the region of 1425 and 1375 cm^−1^ were due to hydroxyl stretching vibration. The band in the region of 1085 cm^−1^ was ascribed to the C−O stretching vibration of KGM. However, with the KGM admixing into cement, the stretch vibration of −COH (around 1085 cm^−1^) of KGM decreases apparently where the in-plane bend vibration of −HOH of KGM (around 1645 cm^−1^) underwent a redshift with the increase in curing age (a redshift of 50 cm was observed after 3 days of curing). This phenomenon was attributed to the chelation interactions between ions in cement and the functional hydroxyl groups on KGM. It also showed that the deacetylation and alkaline hydrolysis of KGM occurred, which could be illustrated by the disappearance of the stretch vibration of −CO (1740 cm^−1^), and the narrowing of the broad peak of −OH (3435 cm^−1^) with the pH value of cement paste increased to a peak value at early hydration periods [24,48]. In other words, KGM would gel in the cement pore solution, which also implied that the film-like substance was probably the product of KGM gelation. Besides, ICP-OES analysis was used to explore the effect of KGM on various ions in the pore solution. As shown in Figure 6b, with the addition of KGM, the concentration of Ca^2+^ decreased sharply, whereas the concentration of Al^3+^ and ${\mathrm{SiO}}_{3}^{2-}$ increased rapidly, which implied that KGM would chelate with Ca^2+^. Insufficient Ca^2+^ content hinders the formation of hydration products, leading to a relative increase in Al^3+^ and ${\mathrm{SiO}}_{3}^{2-}$. As mentioned above, a gelled network of KGM formed on the cement particle surfaces at a high pH value in the early hydration period and chelated with Ca^2+^, which could hinder the water migration and the formation of hydration products.

**Figure 7:**
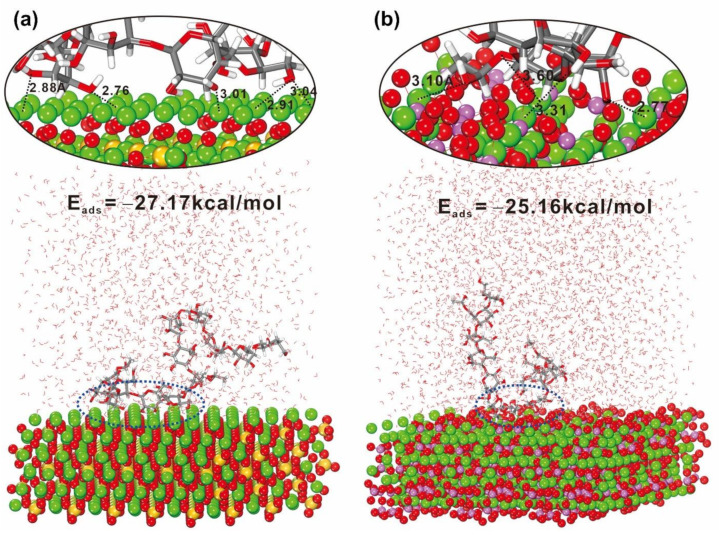
Snapshot of KGM molecule chain interacts with the C_3_S (001) (**a**) and the C_3_A (100) (**b**). Color: grey for carbon; red for oxygen; yellow for silicon, green for calcium, and mauve for aluminum.

Furthermore, molecular dynamics simulations were employed to gain deeper insight into the interaction between KGM and cement clinkers, involving in C_3_A and C_3_S. The adsorption energy (*E_ads_*) between KGM molecular chains and cement clinker surfaces is calculated as *E_ads_* = *E_total_* − *E_surface+water_* − *E_KGM+water_* + *E_water_*, where *E_total_* is the total energy, *E_surface+water_*, and *E_KGM+water_* denote the energy of the system without KGM and without the cement clinker surfaces, respectively. *E_water_* represents the energy of water molecules. The greater absolute value of *E_ads_* indicates the stronger interactions between the KGM and substrates. Figure 7 shows the snapshots of the equilibrate KGM-C_3_A (100) and KGM-C_3_S (001) interaction systems. The *E_ads_* for KGM-C_3_S (001) is −27.17 kcal/mol, while the *E_ads_* for KGM-C_3_A (100) is −25.16 kcal/mol. It indicates that KGM can be stably adsorbed on both C_3_S (001) and C_3_A (100) surfaces. Furthermore, the distance between O atoms of hydroxyl groups on KGM and Ca ions in substrates was found to be about 2.76 to 3.60 Å, which indicates the chelation interactions between them.

In summary, the regulation mechanism of KGM on cement setting and hydration is summarized as having the following synergistic effects: (1) a hydrogel network of KGM formed on the surface of cement particles as a semipermeable membrane; (2) KGM would chelate with Ca^2+^. The synergistic effects significantly hinder the nucleation of hydrated products of cements clinkers and slowing down their growth rate by extending the saturation time of Ca^2+^ in the pore solutions. Nevertheless, the hydration products will slowly nucleate and grow in the space which locates between the clinkers and the adsorbed KGM semipermeable membrane. Because water molecules would pass through the KGM semipermeable membrane through osmosis. The ‘retardation barrier’ is broken after the amount of hydration product is sufficient to break the semipermeable membrane, then the hydration process of cement enters the accelerated period.

## 4. Conclusions

In this study, the influence of KGM on the setting time, fluidity, mechanical properties, and the hydration kinetics of cement are systematically explored, and the mechanism between KGM and cement is proposed by investigating the variation of hydration heat, phase composition, and microstructures of the KGM. Firstly, KGM served as a retarder via delaying the appearance of cement hydration products (especially CH) without the late strength damage, and the retarding effect depended on the KGM molecular weight and dosage where the retardation would be more significant with the increase in KGM content and molecular weight. Secondly, KGM might be a potential TRI, which delays cement hydration while reducing the heat of hydration. The retarding and the temperature increase inhibiting effects of KGM on cement were attributed to the hydrogel networks of KGM formed on the cement particle surfaces at a high pH value in the early hydration period and chelated with Ca^2+^, which could hinder water migration and nucleation, and growth of hydration products. In this study, not only the interaction mechanism between polysaccharides and cement was understood but also a new application field for KGM was discovered.

## Data Availability

The authors declared that they have no conflicts of interest to this study. We declare that we do not have any commercial or associative interest that represents a conflict of interest in connection with the study submitted.

## Supplementary Materials

The following supporting information can be downloaded at: https://www.mdpi.com/article/10.3390/polym14051064/s1, Figure S1: The compressive strength of cement mortar admixed with different KGM dosages with w/c ratio of 0.5 at (a) 3 days, (b) 7 days and (c) 28 days and the porosity of cement mortar admixed with different KGM dosages with w/c ratio of 0.5 at 28 days: (d) the distribution of pore size and (e) cumulative pore volume; Figure S2: Schematic diagram of cement hydration process; Figure S3: (a) The fluidity of cement pastes with different dosages of KGM with w/c ratio of 0.26 and (b) viscosity of cement paste with different KGM contents; Figure S4: FTIR spectrums of the cement pastes admixed with various KGM dosages: (a) pristine OPC and OPC with (b) 0.02 wt %, (c) 0.1 wt %; (d) 0.2 wt % of KGM; Figure S5: The morphology of hydration products changes with 0.2 wt % KGM dosages; Table S1: OPC composition analysis; Table S2: CSA composition analysis; Table S3: CAC composition analysis; Table S4: Molecular weight and apparent viscosity of KGM; Table S5: The influence of various admixtures on cement pastes’ setting time and heat release; Table S6: The influence of different KGM content and molecular weight on cement hydration process and heat release.
