# Supplementary material for: Konjac Glucomannan Induced Retarding Effects on the Early Hydration of Cement
Source: Polymers (Basel). 2022 Mar 7;14(5):1064. doi: 10.3390/polym14051064 (PMC8914915; doi:10.3390/polym14051064)
Supplement: Supplementary file 1 [file polymers-14-01064-s001.zip › polymers-1601925-supplementary.pdf]

Supporting Information

Konjac glucomannan induced retarding effects on the early hydration of cement

Table S1. OPC composition analysis

Phase	CaO	SiO_2	Al_2O_3	SO_3	Fe_2O_3	MgO	K_2O	TiO_2
Content (%)	64.65	20.42	4.68	4.55	2.92	1.08	0.78	0.32
Phase	P_2O_5	Na_2O	SrO	MnO	BaO	Cl	ZnO	ZrO_2
Content (%)	0.24	0.11	0.06	0.05	0.05	0.04	0.03	0.02

Table S2. CSA composition analysis

Phase	CaO	Al_2O_3	SO_3	SiO_2	Fe_2O_3	TiO_2	MgO	F
Content (%)	60.73	15.26	14.21	4.97	2.52	0.87	0.67	0.22
Phase	Na_2O	Cl	P_2O_5	SrO	MnO	BaO	ZrO_2	ZnO
Content (%)	0.15	0.15	0.06	0.05	0.05	0.05	0.04	0.01

Table S3. CAC composition analysis

Phase	Al_2O_3	CaO	SiO_2	TiO_2	Fe_2O_3	SO_3	K_2O	MgO
Content (%)	50.59	36.48	6.11	2.88	2.05	0.64	0.58	0.22
Phase	P_2O_5	ZrO_2	SrO	Cl	Na_2O	ThO_2	Ga_2O_3	Nb_2O_5
Content (%)	0.17	0.10	0.06	0.04	0.04	0.02	0.01	0.01

Table S4. Molecular weight and apparent viscosity of KGM

	Treatment time (min)	Viscosity average molecular weight (10^5 Da)	apparent viscosity (mPa·s)
KGM-1	60	0.8	5800
KGM-2	20	1.1	10600
KGM-3	0	2.8	17650

Table S5. The influence of various admixtures on cement pastes' setting time and heat release

Admixtures	Optimal dosage (wt %)	Retarding time (min)		Temperature rise inhibiting		Refs
		$\Delta\, t_I$ $(= t_{I\,(admixture)} - t_{I\,(OPC)})$	$\Delta\, t_F$ $(= t_{F\,(admixture)} - t_{F\,(OPC)})$	Heat release delay time (h)	$\Delta_{Heat\ release} = \frac{Q_{OPC} - Q_{admixture}}{Q_{OPC}}$	
Sodium Fluosilicate	0.5	580	576	-		[31]
Threitol	1.5	15	30	-		[32]
Xylitol	0.7	325	355	-		
Sorbitol	0.8	335	375	-		
Sucrose	0.15	310	340	-		
Triisopropanolamine	1	-		0.3	+6%	[33]
Glucose	1	-		5	+13%	
Starch	0.1	-		11	+7%	[34]
Zinc oxide / sucrose	0.5/0.15	-		43	+7%	[35]
Triethanolamine	0.1	40	-	0.3	-	[36]
Sodium gluconate	0.24	145	150	40	-	[37,38]
Maltodextrin	0.2	180	480	18	-12%	[12]
KGM	0.2	385	1150	90	-15%	Our work

Table S6. The influence of different KGM content and molecular weight on cement hydration process and heat release.

Fig.	Cement and dosage (wt %) /molecular weight (10^5 Da)	The induction period deadline (h)	The peak exotherm of acceleration (mW/g)	The acceleration period deadline (h)	The cumulative heat (J)
a, e	OPC (0, 0.02, 0.1, 0.2)	3.5, 4.4, 20.0, 70.0	4.4, 4.1, 3.3, 2.8	17.5, 20.8, 48.2, 129.8	644.5, 658.0, 653.2, 566.9
b, f	CAC (0, 0.05, 0.2)	5.5, 10.0, 20.0	11.3, 9.8, 1.7	9.2, 17.0, 26.5	319.5, 309.5, 233.0
c, g	CSA (0, 0.05, 0.2)	2.0, 3.0, 4.0	5.58, 5.54, 3.43	3.2, 4.2, 6.7	243.4, 235.9, 221.0
d, h	OPC (0, 0.8, 1.1, 2.8)	3.5, 5.2, 5.8, 11.0	2.2, 1.6, 1.5, 0.8	16.5, 33.5, 34.8, 48.2	333.3, 325.9, 321.9, 319.4

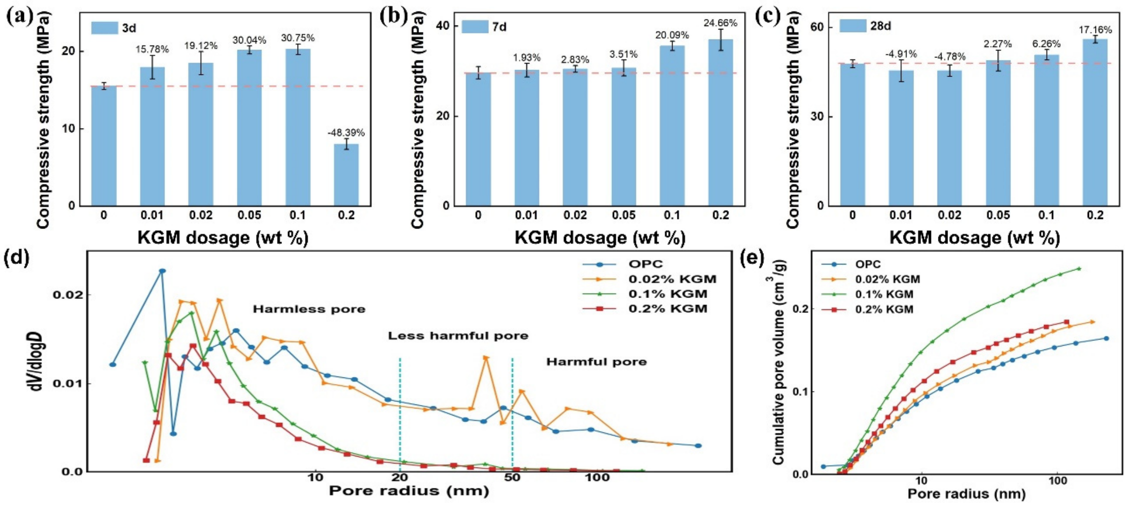

Figure S1 The compressive strength of cement mortar admixed with different KGM dosages with w/c ratio of 0.5 at **(a)** 3 days, **(b)** 7 days and **(c)** 28 days and the porosity of cement mortar admixed with different KGM dosages with w/c ratio of 0.5 at 28 days: **(d)** the distribution of pore size and **(e)** cumulative pore volume.

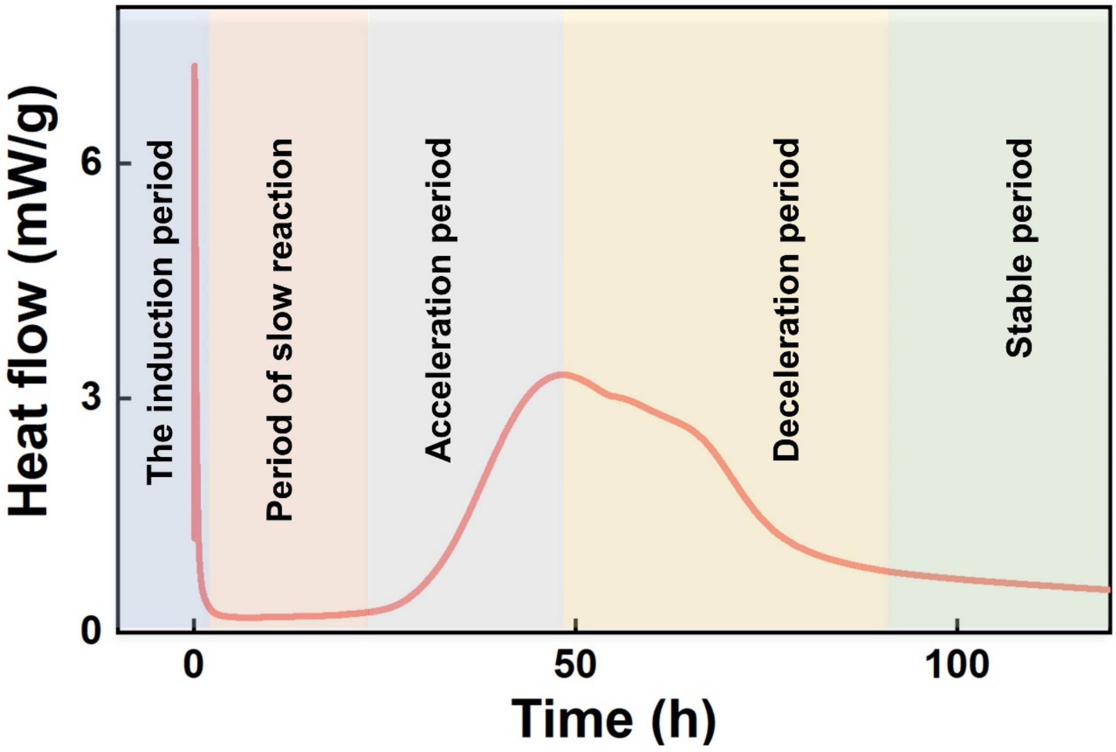

Figure S2 Schematic diagram of cement hydration process.

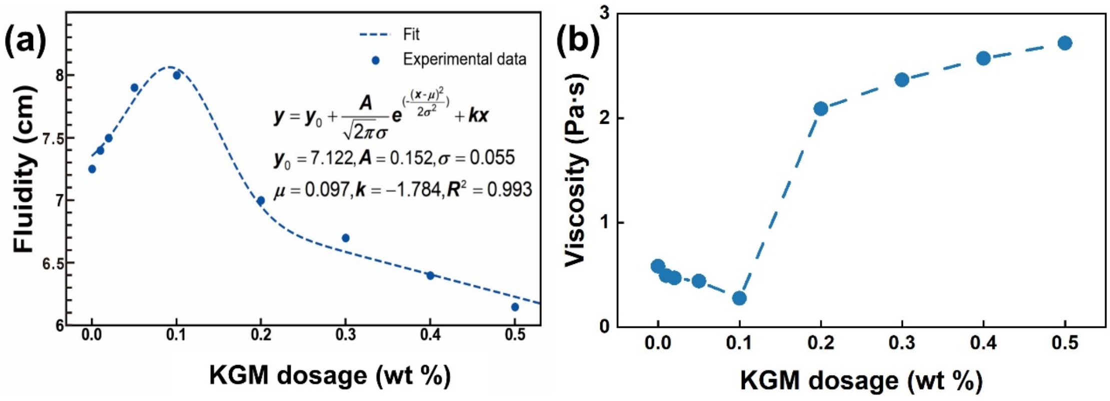

Figure S3 (a) The fluidity of cement pastes with different dosages of KGM with w/c ratio of 0.26 and **(b)** viscosity of cement paste with different KGM contents.

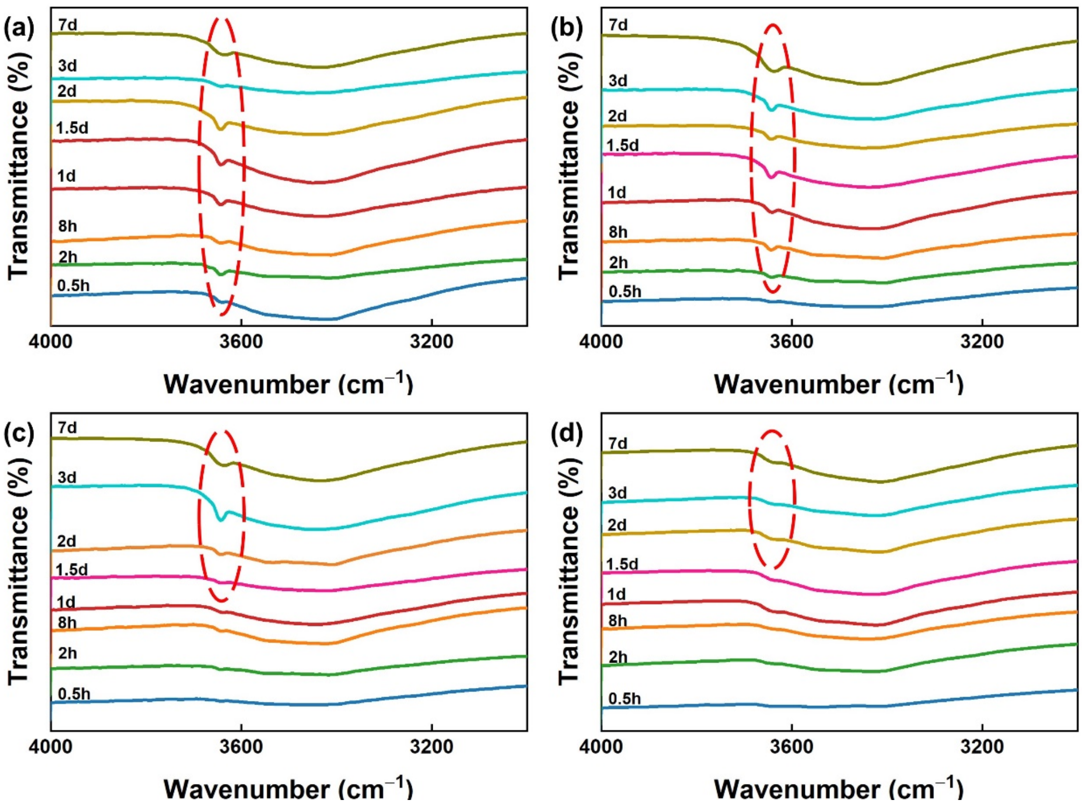

Figure S4 FTIR spectrums of the cement pastes admixed with various KGM dosages: **(a)** pristine OPC and OPC with **(b)** 0.02 wt %, **(c)** 0.1 wt %; **(d)** 0.2 wt % of KGM.

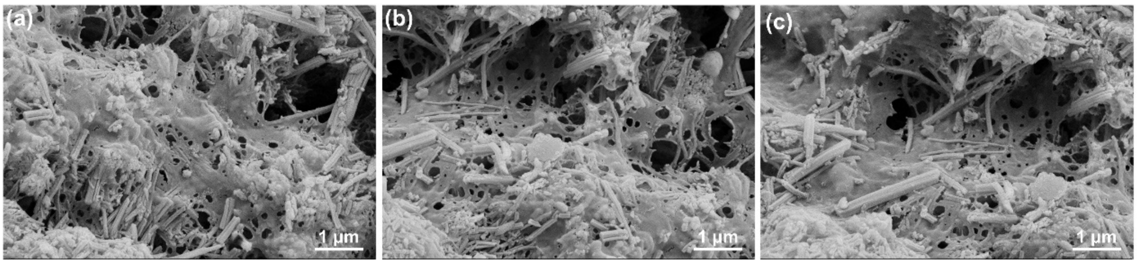

Figure S5 (a-c) The morphology of hydration products changes with 0.2 wt % KGM dosages.
